# Supplementary material for: Event-induced modulation of aperiodic background EEG: Attention-dependent and age-related shifts in E:I balance, and their consequences for behavior
Source: Imaging Neurosci (Camb). 2024 Jan 5;2:imag-2-00054. doi: 10.1162/imag_a_00054 (PMC12235562; doi:10.1162/imag_a_00054)
Supplement: Supplementary Material [file imag_a_00054-supp.pdf]

Supplementary materials

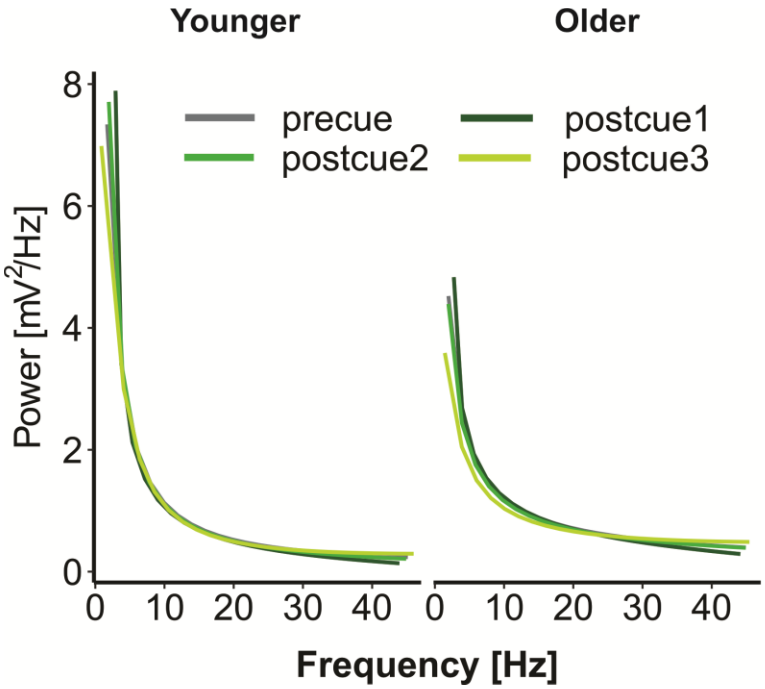

Figure 1S. Average aperiodic component by time window and age group. Dark, medium, and light green, as well as gray, denote the post-cue-1, post-cue-2, post-cue-3, and pre-cue time windows, respectively, all following ERP removal.
